# Deep brain stimulation in Parkinson’s disease

**DOI:** 10.1186/2047-9158-2-22

**Published:** 2013-11-18

**Authors:** Raja Mehanna, Eugene C Lai

**Affiliations:** 1 UT Move, University of Texas Health Care Science Center, Houston, TX, USA; 2 Department of Neurology, Houston Methodist Neurological Institute, 6560 Fannin, Suite 802, Houston 77030, TX, USA

**Keywords:** Deep brain stimulation, Parkinson’s disease, Subthalamic nucleus, Globus pallidus

## Abstract

For the last 50 years, levodopa has been the cornerstone of Parkinson’s disease management. However, a majority of patients develop motor complications a few years after therapy onset. Deep brain stimulation has been approved by the FDA as an adjunctive treatment in Parkinson disease, especially aimed at controlling these complications. However, the exact mechanism of action of deep brain stimulation, the best nucleus to target as well as the best timing for surgery are still debatable. We here provide an in-depth and critical review of the current literature on this topic.

## Introduction

Parkinson’s disease (PD) is a neurodegenerative disorder characterized by the clinical tetrad of motor dysfunction, including tremor, rigidity, akinesia (or bradykinesia) and postural instability (TRAP). It has a prevalence of 1 to 2% above the age of 60 years [1] and typically develops between the ages of 55 and 65 years. The tremor typically starts asymmetrically and is present at rest, usually involving hands, legs, jaw, lips, but sparing the head. Pathologically, PD is classified as a synucleinopathy, associated predominantly with the loss of dopaminergic neurons in the substantia nigra, but other brainstem neurons have been found to degenerate in PD, possibly contributing to not only motor but also non-motor impairment [2]. Indeed, PD is now considered to be a complex syndrome, and is no longer regarded as a pure motor system disorder [3]. For example, neurobehavioral abnormalities are frequent in advanced PD and include depression, dementia, bradyphrenia, apathy, fearfulness, anxiety, emotional lability, social withdrawal, visual–spatial impairment, sleep disturbance and psychosis. Autonomic involvement leads to constipation, bladder and sphincter dysfunction and orthostatic hypotension. Dermatological problems such as seborrhea, sensory problems such as pain and tingling, and special sense disorders such as hyposmia and vestibular dysfunction are also well described [3].

Five years after initiation of therapy, a majority of patients develop medication related motor complications, namely levodopa induced dyskinesias (LID) and motor fluctuations. LID are choreic, stereotypic, and dystonic movements affecting any part of the body [2] and occurring either at peak dose or when the medication is kicking in or wearing off (dyskinesia-improvement-dyskinesia effect). Motor fluctuations occur when the duration of each medication dose is too short and the symptoms of PD recur sooner that initially. This weaning off the medication effect can occur suddenly and unexpectedly, leaving the patient with markedly decreased mobility and/or severe tremor [4,5]. Deep brain stimulation (DBS) has been developed primarily to address these treatment related motor complications and therapeutic failures.

The estimated annual health care cost of PD ranges from $2,000 to more than $20,000 per patient [6], with an estimated global economic burden of $4.63 billion [7], the greatest part of which being represented by nursing homes and personal care-giving [8-10] which are much higher in the later stage PD [11].

## Brief overview of the motor circuitry of the basal ganglia

To better understand the hypothesized mechanism of action of DBS in PD, we will first briefly review the motor circuitry of the basal ganglia. Different motor and non-motor cortical areas project primarily to the striatum which has two major projections: the direct pathway to the globus pallidus pars interna (GPi) and the indirect projection to the GPi via the globus pallidus pars externa and the subthalamic nucleus (STN). The GPi serves as the major output nucleus, which connects back to the cortex via the thalamus. Modulated by the substantia nigra pars compacta, the indirect pathway exerts surround inhibition and thus facilitates an excitatory drive to muscles responsible for a given movement and suppresses unwanted motor activity not relevant to the primary movement. Thus, PD is thought to result from over-activation of the indirect pathway leading to an increased output from the GPi and a decrease in spontaneous movement [12]. This model of the basal ganglia and its connections is, of course, an oversimplification of a complex network that, when disrupted, can result in a range of motor abnormalities [13]. For example, a hyperdirect pathway, projecting directly from the cortex to the STN, and from there to the GPi has recently been added to this model [14].

## Pathophysiology of PD

The loss of dopaminergic neurons in the substantia nigra, the main functional characteristic of PD, affects the circuit described above and leads to the cardinal motor symptoms of PD. While the exact mechanism of this process is unknown, animal research as well as human recordings have provided functional and biochemical evidence that bradykinesia in PD results from excessive activity in the STN and the GPi [3,15-17]. This leads to an exaggerated beta (10-30 Hz) synchronization within and between structures in the basal ganglia circuitry [3] that could also contribute to rigidity and akinesia [18,19].

The pathophysiology of rest tremor in PD is less clear and probably more complicated. The existence of a unique pacemaker driving the tremor in the thalamus or the GPi has been suggested and then rejected [3]. This symptom most likely results from a dysfunction of both the striato-pallidal-thalamocortical and the cerebello-dentato-thalamocortical circuits [20], with hyperactivity and hypersynchronization between central oscillators [21]. However, contrary to bradykinesia, tremor does not seem to be dependent on beta oscillatory synchronization [22,23].

## Possible mechanism of action of DBS

DBS acts through delivering an electrical current in a specific target area of the brain. This current can be modulated through modification of voltage, frequency and duration of each electrical pulse delivered. The delivered energy creates an electrical field of variable size and shape according to the parameters used for stimulation. Although initially believed to stimulate the target, thus the name of the whole process, it seems that DBS actually excites the neuronal fibers, but inhibits the neural cells [24,25]. In fact, GPi DBS decreases the GPi mean firing rate back to a normal range in animal models as well as PD patients [26], and high frequency DBS has a similar effect as dopamine replacement therapies, and promotes faster (about 70 Hz) non-hypersynchronous activity in the basal ganglia, correlated with clinical improvement [27-30]. This might be achieved through stimulation of bypassing inhibitory pathways, synaptic inhibition, depolarizing blockade, synaptic depression, and simulation-induced disruption of pathological network activity [26,31]. Overall, this leads to modifications of the firing rate and pattern of neurons [32] in the basal ganglia, as well as local release of neurotransmitters such as glutamate and adenosine [33-35]. In addition, it seems that DBS also increases blood flow and stimulates neurogenesis [36].

Over the last few years, functional imaging, specifically functional magnetic resonance imaging (fMRI), positron emission tomography (PET) and single-photon emission computed tomography (SPECT), has been used in an attempt to clarify the mechanism of action of DBS. In fMRI, blood-oxygen-level-dependent (BOLD) signals are acquired, and oxygenated blood marks areas of neural stimulation or inhibition [37-39]. On the other hand, PET and SPECT allow for imaging of multiple activity markers, such as blood flow, glucose and oxygen metabolism [40,41]. While fMRI is less powerful than nuclear medicine techniques, it provides a much better spatial and temporal resolution.

Because of the suspected inhibitory DBS effects in electrophysiological studies, reduced STN blood flow or glucose metabolism would have been expected on functional imaging. However, the opposite has been found to be true in an overwhelming majority of imaging studies to date [42-49]. In addition, BOLD activation in the area surrounding the electrode has been reported [48,49], despite the electrode imaging artifact preventing direct observation of the STN around the electrode. This discrepancy between apparent STN inhibition in single-cell studies and activation in imaging studies might be explained by a few hypotheses [50]. First, electrophysiological recordings identify short neuronal modulation (in the order of milliseconds) while neuroimaging methods may reflect the summed activity changes over seconds to minutes. Second, non-neuronal contributions to the change in blood flow and/or glucose metabolism cannot be excluded, and could confound the results of neuroimaging. Finally, it is possible that PET and fMRI actually detect the increased activity in the axons, rather than in the cell bodies. Complicating matters further, some imaging studies after STN DBS have showed increased activity in the GPi [47,48,51] while others reported decreased activity in that nucleus [42,52].

In summary, it is still unclear how exactly DBS affects the firing rate and pattern of neurons and how these changes actually modify the symptoms of Parkinson’s disease. DBS is presently more of an empirically proven treatment in search of physiological explanation.

## Clinical aspect

The effect of DBS on the cardinal symptoms of PD have been established in three randomized controlled clinical trials (Table 1) [53-55].

**Table 1:** Randomized controlled trial comparing DBS to optimal medical management

**Author, year**	**Number of patients**	**Follow up**	**Target**	**Results (primary outcome)**
Deuschl et al., 2006 [53]	156	6 months	BL STN	-Quality of life better with DBS
				-Motor symptoms better with DBS.
Weaver et al., 2009 [54]	255	6 months	BL STN or GPi	Dyskinesia- free ON time 4.6 hours longer with DBS
Williams et al., 2010 [55]	366	12 months	BL STN or GPi	-Quality of life better with DBS

***Legend:*** *BL: bilateral; STN: subthalamic nucleus; GPi: Globus pallidum pars interna; DBS: deep brain stimulation.*

The first trial was conducted in Germany and Austria on 156 patients with PD and persistent motor symptoms despite optimal medical therapy [53]. These patients were randomly assigned to DBS of the STN (STN DBS) or optimal medical management. DBS assigned patients had a statistically better quality of life, as measured by the Parkinson’s Disease Questionnaire-39 (PDQ-39), as well as motor symptoms control, as measured by the motor part of the Unified Parkinson’s Disease Rating Scale (UPDRS, Part III). Another trial conducted in the United States as a double-blinded randomized study and involving 255 patients showed that DBS in either the STN or GPi increased the amount of dyskinesia free ON time at 6 months by 4.6 hours compared to the optimally managed medical group [54]. The statistically significant improvement in quality of life after DBS compared to optimal medical management was reproduced in a third trial on 366 patients followed for 1 year in the United Kingdom [55].

These and other studies have also shown that DBS typically improves “off” time by 4 to 6 hours per day [54,56,57], and reduces off symptoms by 60% and medication induced dyskinesias in STN DBS by 60-80% [58]. In addition to the improvement of motor symptoms, DBS can also improve pain, emotion, akathisia and even autonomic function as reported in 3 of 11 patients [58-63]. In some studies, working memory and psychomotor speed improved in the ON versus OFF stimulation state of bilateral STN DBS [64]. Open label studies and prospective series also suggest that bilateral as well as unilateral STN DBS improves sleep quality and increases total sleep time, likely by allowing a better control of PD symptoms at night and by a direct effect on sleep architecture (Table 2) [65-67].

**Table 2:** Summary of benefits and side effects of DBS

**Reported benefits**	**Reported side effects**
Improved QOL	Wound/hardware infection
Decreased OFF time	Intra cranial hemorrhage
Reduced OFF symptoms	Post-operative seizures
Reduced LID	Muscle twitches or tonic contractions
Improved sleep	Paresthesia
Can also improve:	Dysphagia
pain,	Cognitive impairment
anxiety	Speech impairment
emotion	Visual complaints
akathisia	Mood disorders
autonomic function	Anxiety
working memory	Apathy
psychomotor speed	Impulse control disorders,
	Obsessive-compulsive disorder
	Aggression
	Weight gain

**Legend**: *DBS: deep brain stimulation; QOL: quality of life; LID: levodopa induced dyskinesias.*

DBS is a reasonable option to consider when medication adjustments do not control disabling symptoms anymore [6,66,68]. However, not all such PD patients are candidates for DBS, and a thorough multidisciplinary screening process is required to determine those who are good candidates [69,70]. In the current context of reducing unnecessary expenses, this screening process can be staged to help reduce costs and improve its efficiency. Patients should first undergo a thorough clinical evaluation from the neurologist to ascertain the diagnosis and exclude other causes of parkinsonism, such as vascular parkinsonism or Parkinson Plus Syndromes that are refractory to DBS. The neurologist should optimize medical management before offering DBS. Patients should then discuss at length with the neurologist and neurosurgeon to assess their understanding of the risk/benefits ratio of the surgery. If the patients then clearly understand this commitment and wish to proceed, the ON levodopa/OFF levodopa motor testing can be performed. In the ON/OFF testing, the patient is asked to withhold all PD medications for 12 hours before undergoing examination with the UPDRS. The patient is then given his regular dose of levodopa and the UPDRS is administered again after the patient reports feeling the full effect of this dose. ON and OFF UPDRS scores are compared and an improvement of at least 30% after medication intake is typically recommended to proceed to the next step. Indeed, only the symptoms that improve with levodopa are expected to improve with DBS, with the notable exception of medication refractory tremor that can still improve after surgery. DBS should thus be offered to patients with levodopa-responsive symptoms [71]. At that point, if the patient is still considered a good candidate for surgery, brain MRI, neuropsychological testing and psychiatry evaluation would be pursued.

Animal models of PD have suggested that STN DBS might prevent further neuronal loss and thus have a neuroprotective effect [72-77]. The mechanisms of such effect are unknown, but could include a reduction in excitotoxic induced damage [76]. However, these results have not yet been reproduced in the few human studies evaluating DBS for a neuroprotective effect [78,79].

Similar to the medical management of PD, DBS is thus a symptomatic treatment and has not been shown to slow the progression of the disease. The duration of the therapeutic benefit has not been clearly established, but modifying the parameters of the stimulation allows the healthcare provider to tailor delivered energy to the patient’s symptoms and evolution, and sustained clinical improvement had been reported up to 10 years after implant [80-84]. However, the greatest sources of disability in late-stage PD, including drug-resistant axial motor features and non-motor symptoms, especially psychiatric disorders and cognitive decline, are not significantly modified by DBS [85].

Occasionally, patients might have a suboptimal response to DBS. In a series of 41 such patients, 31 of whom initially diagnosed with PD, Okun et al. [86] reported poor patient screening and selection as the main causes for DBS failure. Other causes included improper electrode location as well as suboptimal DBS programming and medication management. 51% of these patients markedly improved with appropriate management adjustments [86]. Occasionally, patients might require surgical lead repositioning or even the addition of another lead in another brain structure [87].

While DBS related expenses such as the hardware, surgery, post-surgical programming visits and personal care-giving are relevant [88], STN DBS seems economically more beneficial than optimal medical management through a decrease in drug requirement and cost as well as a reduction in nursing care cost [58,89-91]. No such data exist for GPi DBS.

## Side effects

Like all surgeries, DBS implant is not deprived of potential complications (Table 2). The rate of post-operative wound or hardware infection varies from 1.2% to 15.2% in different series [92-94], and most often such incidences require removal of the hardware in addition to an antibiotic course [6,94]. Intracranial hemorrhage has been reported in 5% of cases, but was symptomatic only in 2.1% of implanted patients and caused a permanent deficit or death in 1.1% [95]. Another larger study reported 1% of intracranial hemorrhage and stroke respectively, in 299 patients after DBS placement [57]. Older age and a history of hypertension have been associated with an increased risk of hemorrhage [95]. Post-operative seizures were reported in 2.4% of patients in one review [96], but these do not increase the risk of epilepsy. Prophylactic anticonvulsive therapy before and around surgery is currently unjustified.

Because the energy delivered to the target can spread to nearby structures and alter their function as well, DBS can induce cognitive and speech impairment, visual complaints, mood disorders including suicidal ideation [97,98], dysphagia, paresthesia and muscle twitches or tonic contractions. These are most frequently due to a lead positioned too close to the medial STN, optic tract, oculomotor nerve, internal capsule or lemniscal tract. Ideally, these side effects could be reduced or avoided by optimal placement of the lead, with a combined use of anatomic localization and intraoperative microelectrode recording. In addition, using bipolar stimulation settings will create a smaller field with less spreading to adjacent structures, and consequently fewer side effects. The most frequent cognitive side effect to DBS seems to be a decrease in verbal fluency, especially when the STN is targeted [99], although some authors argue this to be a consequence of the implant rather than the stimulation [6]. In a trial comparing 123 patients randomized to bilateral STN DBS or optimal medical management with neuropsychological and psychiatric evaluations at baseline and at 6 months, there was a selective decrease in frontal cognitive function, especially verbal fluency and naming in the DBS group. However, overall cognition was preserved and anxiety was more improved in that group [100]. Other reported neuropsychiatric adverse effects include anxiety, apathy [101,102], decreased frontal cognitive function [100], decreased executive function [103], impulse control disorders, obsessive-compulsive disorder and aggression [104-108]. Poor pre-operative affective state may predict continued depression post DBS [109], highlighting the need for appropriate pre-surgical patient screening. Finally, DBS can also lead to weight gain [110].

These side effects are more frequent in bilateral procedures [111], and a unilateral implantation can also have bilateral benefits [111-113]. The decision to implant unilaterally or bilaterally needs to be tailored to the patient’s needs.

## Target selection

Once a patient is determined to be a good candidate for DBS, a target for the procedure has to be selected. Ablative surgery or DBS of the ventral intermediate (Vim) nucleus of the thalamus is being used for essential and other secondary causes of tremor. However, because it does not address the other cardinal motor symptoms of PD, Vim DBS is rarely used for that disorder [114]. The two main targets considered for DBS in PD are the STN and the GPi (Table 3).

**Table 3:** Randomized controlled trials comparing STN and GPi DBS

**Author, year**	**Number of patients**	**Follow up**	**Side**	**Similar results**	**Differences in results**
Anderson et al., 2005 [115]	20	12 months	Bilateral	-Motor symptoms	-Greater decrease in dopaminergic drug dosage with STN.
					-Cognitive and behavioral complications exclusively with STN.
Okun et al., 2009 [116]	45	7 months	Unilateral	-Motor symptoms.	-Worse verbal fluency with STN.
				-Side effects including mood and cognition.	-Greater improvement in QOL with GPi [117].
					-Higher risk to require controlateral DBS implant in STN group [112].
Follett et al., 2010 [57]	299	24 months	Bilateral	-Motor symptoms.	-Greater decrease in dopaminergic drug dosage with STN.
				-Side effects profile.	-Worse decline in visuomotor processing with STN
					-Depression improved with GPi but worsened with STN.
Weaver et al., 2012 [118]	159	36 months	Bilateral	-Motor symptoms.	-Greater decrease in dopaminergic drug with STN.
				-Side effects profile.	-Worse cognitive performance with STN
Odekerken et al., 2013 [119]	128	12 months	Bilateral	-Quality of life.	-Greater decrease in dopaminergic drug dosage with STN.
				-Cognitive, psychiatric and behavioral side effects	-Greater improvement in the OFF phase motor score with STN
					- Greater improvement in disability with STN

***Legend:*** *STN: subthalamic nucleus; GPi: Globus pallidum pars interna; DBS: deep brain stimulation; QOL: quality of life.*

Anderson et al. [115] conducted the first randomized controlled trial comparing STN DBS and GPi DBS. This trial included only 20 patients followed for 12 months and showed both targets to be equally effective for improving PD motor symptoms and dyskinesia. It also showed a greater decrease in dopaminergic medication use in the STN group (p = 0.08) as well as cognitive and behavioral complications exclusively in that group.

The COMPARE trial [116] included 45 patients with unilateral GPI or STN DBS who were followed for 7 months. The 2 targets were similar in motor control improvement and side effects profiles including mood and cognition, except for worsening of verbal fluency in the STN DBS group. However, GPi DBS patients had a bigger improvement in their quality of life compared to STN patients (38 vs. 14%, respectively; P = 0.03) with decrease in verbal fluency potentially contributing to less improvement in the STN patients [117]. In a follow up to the COMPARE trial including 52 patients, unilateral STN DBS implant carried a 5.2 times increased risk to require a contralateral DBS implant at 3 years, when compared to unilateral GPi DBS implant [112].

In a large multicenter randomized control trial, Follett et al. [57] followed 299 patients for 2 years. GPi and STN DBS were similar in motor control improvement and side effects profiles, except for more severe decline in visuomotor processing as well as requiring lower doses of dopaminergic medications in STN DBS patients (p = 0.03 and 0.02 respectively). In addition, the level of depression improved in the GPi DBS group while it worsened in STN DBS patients (p = 0.02). At the 3-year follow-up of the same group, including 159 patients, Weaver et al. [118] showed that the 2 targets were similar in motor control improvement and side-effect profiles, except for worse cognitive performance in the STN group at 3 years (p = 0.01). This study also confirmed the greater reduction in dopaminergic drugs in the STN group.

The most recent double blinded randomized controlled trial comparing STN and GPi DBS [119] included 128 patients and reproduced the lack of significant difference in quality of life improvement between the 2 targets at 1 year. However, there was greater improvement in the OFF phase motor score and disability in STN DBS patients (p = 0.03). This study also confirmed a greater decrease in dopaminergic medication doses in the STN group (p = 0.01). However, there was no difference in cognitive, psychiatric and behavioral side-effects between the 2 groups.

It is unclear why STN DBS might have a higher rate of cognitive decline and/or depression in some studies. These finding first need to be reproduced, but both current spreading to limbic regions as well as the decreased dose of dopaminergic drugs might be contributory [120]. Taking all these data into consideration, the current tendency is to prefer targeting the STN because of a greater improvement in the OFF phase motor symptoms as well as a higher chance to decrease the medication dosage and a lower battery consumption linked to the use of lower voltage in the STN compared to the GPi DBS. Lower battery consumption correlates with longer battery life and thus less frequent surgery to replace it [119]. On the other hand, GPi DBS has a direct anti-dyskinestic effect when stimulation is delivered to the ventral part of the nucleus, while decrease in LID after STN DBS can be achieved through the decrease in medication dose. For that reason, GPi can be the preferred target if LID is the main complaint. As most studies showed that STN DBS resulted in more cognitive and behavioral deficits [57,115,116,118], GPi DBS might be preferred for patients with mild cognitive impairment and psychiatric symptoms.

Because of its role in locomotion, the pedunculopontine nucleus (PPN) has been suggested as a DBS target to address gait difficulty and freezing of gait, which are typically resistant to STN or GPi DBS. A few small observational or open labeled studies have been conducted, evaluating PPN DBS as an add-on to STN DBS [82,121-126]. Precise anatomical lead locations as well as results were controversial, and PPN DBS cannot be recommended at this point.

## Timing of the surgery

Currently, patients are not considered for DBS unless they do not tolerate levodopa, become resistant to medications or have complications from medical therapy, while still being medically and psychologically fit for surgery. Some authors suggest that exhausting all pharmacological options before considering DBS can delay the surgery to a time when the patient is no longer fit because of disease progression [58,127,128].

A small randomized trial of bilateral STN DBS in 10 patients with early PD (mean duration of 7 years) compared to 10 matched medically treated controls [129] showed a significant benefit from DBS on quality of life, motor control and decrease in levodopa doses. More recently, Schuepbach et al. [130] conducted a randomized controlled trial comparing bilateral STN DBS to optimal medical management in 251 PD patients aged less than 60 years, with PD for more than 4 years but with motor fluctuations and dyskinesia for 3 years or less. This study demonstrated improvement in quality of life, motor control, as well as mood at 24 months in the DBS group compared to the medical group. Mild side effects were more frequent in the DBS group, but the incidence of moderate to severe side effect was comparable between the 2 groups. With a mean age of onset of 52 years as compared with 59 to 62 years in other trials [55-57,80,119] and a mean duration of PD of 7.5 years, as compared with 10.8 to 13.8 years [53,55-57,119], this study demonstrated the added benefit of early DBS in PD symptoms control, when medical treatment is still effective. However, these results have yet to be confirmed.

Another ongoing trial comparing DBS early in the course of PD to optimal medical management, EARLYSTIM [58,131], involves 247 patients implanted a mean of 7.5 years after diagnosis and within 3 years of development of treatment induced motor complications. Preliminary results report a mean age of 52 years, levodopa therapy duration of 4.9 years and fluctuations and/or dyskinesias present for 1.5 or 1.7 years respectively. At 2 years follow up, quality of life and motor control were superior in the DBS group. Side effects were more frequent in the medical group, and surgical complications resolved entirely.

In addition, an analytic model of STN DBS, defined by off time and applied at an early versus delayed stage, concluded that early DBS increases quality-adjusted life years and reduces treatment costs [132]. However, further studies in this area are warranted before recommending early DBS placement in PD patients. In addition, it would be wise to wait 5 years after the onset of symptoms before performing the surgery, in order to rule out any Parkinson Plus Syndrome.

## Conclusion

For the last 50 years, levodopa has been the cornerstone of PD management. However, a majority of patients develop motor fluctuations and/or LID about 5 years after the initiation of therapy. DBS of the STN or the GPi grant to patients with PD improved quality of life and decreased motor complications, and has been approved as such by the Food and Drug Administration in the US in 2002. We reviewed the experience and available literature on DBS for Parkinson’s disease over the last decade and arrive at the following suggestions. (1) The success of DBS surgery depends on the accurate placement of the leads and meticulous programming of the stimulation. Therefore, it is best accomplished by an experienced team of neurosurgeon, neurologist, and support staff dedicated to the treatment. (2) Reports of surgical complication rates and long-term side-effects of DBS are very variable, so benefits and potential adverse results should not be under- or over-emphasized. (3) While essentially equal in improving the motor symptoms of PD, STN and GPi might have their own benefits and risks, and the choice of the target should be individualized and adapted to the patient’s situation. (4) Knowledge to further improve DBS treatment for Parkinson’s disease, such as a more scientific and reliable protocol on programming, strategies to minimize cognitive and psychiatric complications, and the better long-term maintenance of the implanted device, are still lacking. (5) Data on the impact of DBS on non-motor symptoms affecting the quality of life of PD patients, such as pain, speech or gastro-intestinal complaints, are still scarce. Further research in these areas will help make this useful treatment even more beneficial.

## Competing interests

R Mehanna does not report any conflict of interest.

## Authors’ contributions

RM: conception and design, review of the literature, writing of the initial manuscript, approval of the final form to be published. EL: conception and design, review and critique, approval of the final form to be published.
